# PLP1 may serve as a potential diagnostic biomarker of uterine fibroids

**DOI:** 10.3389/fgene.2022.1045395

**Published:** 2022-10-31

**Authors:** Lei Cai, Zhiqi Liao, Shiyu Li, Ruxing Wu, Jie Li, Fang Ren, Hanwang Zhang

**Affiliations:** ^1^ Reproductive Medicine Center, Tongji Hospital, Tongji Medical College, Huazhong University of Science and Technology, Wuhan, China; ^2^ Institute of Digestive Disease and Department of Medicine and Therapeutics, State Key Laboratory of Digestive Diseases, Li Ka Shing Institute of Health Sciences, The Chinese University of Hong Kong, Hong Kong, China; ^3^ Department of Gynecology, First Affiliated Hospital of Zhengzhou University, Zhengzhou, China

**Keywords:** uterine fibroids, bioinformatics analysis, DNA methylation, PLP1, biomarker

## Abstract

**Objective:** We aim to identify the crucial genes or potential biomarkers associated with uterine fibroids (UFs), which may provide clinicians with evidence about the diagnostic biomarker of UFs and reveal the mechanism of its progression.

**Methods:** The gene expression and genome-wide DNA methylation profiles were obtained from Gene Expression Omnibus database (GEO). GSE45189, GSE31699, and GSE593 datasets were included. GEO2R and Venn diagrams were used to analyze the differentially expressed genes (DEGs) and extract the hub genes. Gene Ontology (GO) analysis was performed by the online tool Database for Annotation, Visualization, and Integrated Discovery (DAVID). The mRNA and protein expression of hub genes were validated by RT-qPCR, western blot, and immunohistochemistry. The receiver operating characteristic (ROC) curve was used to evaluate the diagnostic value.

**Results:** We detected 22 DEGs between UFs and normal myometrium, which were enriched in cell maturation, apoptotic process, hypoxia, protein binding, and cytoplasm for cell composition. By finding the intersection of the data between differentially expressed mRNA and DNA methylation profiles, 3 hub genes were identified, including transmembrane 4 L six family member 1 (TM4SF1), TNF superfamily member 10 (TNFSF10), and proteolipid protein 1 (PLP1). PLP1 was validated to be up-regulated significantly in UFs both at mRNA and protein levels. The area under the ROC curve (AUC) of PLP1 was 0.956, with a sensitivity of 79.2% and a specificity of 100%. Conclusion: Overall, our results indicate that PLP1 may be a potential diagnostic biomarker for uterine fibroids.

## 1 Introduction

Uterine fibroids (UFs) are one of the most common uterine benign neoplasms in women of reproductive age, with a morbidity of 77% (Stewart, 2005), and symptomatic lesions occur in 20%–40% of UFs patients (Leyland et al., 2022). The main clinical symptom includes menorrhagia, abnormal uterine bleeding, infertility, recurrent spontaneous abortion, and other pelvic disorder (Styer and Rueda, 2016; Dolmans et al., 2021). Moreover, UFs are the primary incidents of hysterectomy (Ciarmela et al., 2022) with a quantifiable economic and social burden (Cardozo et al., 2012). Ultrasound is the first-line imaging technique in the evaluation of UFs (Russo et al., 2022). It can provide information about some characteristics of morphology, such as cystic area, echogenicity, borders, and vascularization of the lesion. Nevertheless, it is difficult for clinicians to differentiate the benign myoma in the uterine from malignant leiomyosarcoma accurately. Recently, a novel diagnosis strategy has emerged that integrates the histological features and molecular biomarkers to provide a comprehensive assessment of UFs and determine whether a complete hysterectomy is required (Levy et al., 2013; Trovik et al., 2014; Croce and Chibon, 2021; Machado-Lopez et al., 2021). However, these potential biomarkers still lack reliable clinical utility (Levy et al., 2013), as the sensitivity or specificity of them is less than 75% or 99.6%, respectively (Anderson et al., 2010). Thus, more valuable biomarkers validated for the diagnosis of UFs are desperately required. It may also enable us better understand the mechanism of progression and some important features of UFs.

DNA methylation, one of the epigenetic modifications of DNA in mammalians, refers to the transfer of a methyl group to the fifth carbon of a cytosine residue on the DNA sequence to form 5-methylcytosine (Reik et al., 2001). It occurs in CpG dinucleotides that are clustered frequently in regions of about 1–2 kb in length, called CpG islands, in or near the promoter and first exon regions of genes (Jones, 2012; Schübeler, 2015; Dor and Cedar, 2018). The frequency of CpG in gene regulatory regions is different. It was demonstrated that in leiomyomas, CpG sites were hypomethylated in the distal region of the estrogen receptor-alpha (ER-alpha) promoter combined with the higher ER-alpha mRNA levels (Asada et al., 2008). Besides, the aberrant expression of methyltransferases (Li et al., 2003) and other existence of differently methylated genomic locus in fibroids were also reported to separate the UFs from myometrium (Croce and Chibon, 2015; Braný et al., 2019; Liu et al., 2019; Sato et al., 2019; Maekawa et al., 2022). Based on the specific hypomethylated/hypermethylated genes (Islam et al., 2013; Sato et al., 2016) and the genome-wide DNA methylation profiles of UFs (Navarro et al., 2012; Maekawa et al., 2013), DNA methylation is considered to be the mainstay epigenetic mechanism of UFs. It is involved in the developmental processes of UFs by silencing, switching, and stabilizing genes. Hence, genes associated with DNA methylation may offer us some useful clinical diagnostic biomarkers for UFs. Nevertheless, the hub gene is still unclear.

In the present study, three Gene Expression Omnibus (GEO) datasets were utilized for analyzing the key gene relevant to DNA methylation in UFs. The hub gene was further validated by RT-qPCR, western blot, and immunohistochemistry. Finally, the receiver operating characteristic (ROC) curve was used to evaluate the performance of this biomarker for diagnosing UFs.

## 2 Methods

### 2.1 Obtaining the gene expression profiles in UFs

All three gene expression profiles in leiomyoma and normal myometrium tissue (GSE45189, GSE31699, and GSE593) were obtained from the National Center of Biotechnology Information (NCBI) Gene Expression Omnibus (GEO). The retrieval strategy was present with several keywords: leiomyoma, myometrium, gene expression profiling, and DNA methylation genome-wide association study. The inclusion was as follows: 1) a case-control research design; 2) includes UFs and normal myometrium tissue; 3) The original profiles should contain a genome-wide assessment. The exclusion criteria were the following: 1) non-case–control research design; 2) Other tissue. The analysis of the GSE45189 data set was based on 3 frozen UFs and 3 normal myometrium tissue obtained from the uterus with leiomyoma. The GSE31699 data set includes the gene expression profile of 68 UFs and paired normal myometrium tissue. The GSE593 data set included 6 tissue samples for DEGs analysis only. All details of sample information and experiment type are shown in Table 1.

**TABLE 1:** Gene Expression Omnibus (GEO) data set.

GEO accession	Platform	UFs	Normal myometrium	Experiment type
GSE593	GPL96	5	5	Affymetrix Human Genome U133A Array
GSE45189	GPL6244	3	3	Affymetrix Human Gene 1.0 ST Array
	GPL13534	3	3	HumanMethylation450 BeadChip
GSE31699	GPL6947	68	68	Illumina HumanHT-12 V3.0 expression Beadchip
	GPL8490	68	68	Illumina HumanMethylation27 BeadChip

Note: UFs, uterine fibroids.

### 2.2 Identification of DEGs between uterine fibroids and normal myometrium

We analyzed the DEGs between UFs and normal myometrium tissue from the gene expression profiles of GSE45189, GSE593, and GSE31699 datasets respectively. The differential DNA methylation genes were analyzed from the genome-wide DNA methylation profiles in GSE45189 and GSE31699 datasets respectively. All differential genes were identified using the online analysis tool GEO2R (https://www.ncbi.nlm.nih.gov/geo/geo2r/). Benjamin-Hochberg was applied for the control of false discovery rate (FDR), and *p* < 0.05 was utilized as the database’s cut-off criteria. We draw the Venn diagram by the online tool (http://bioinformatics.psb.ugent.be/webtools/Venn/).

### 2.3 Gene Ontology analysis

In this study, Gene Ontology (GO) analysis was performed by the online tool, Database for Annotation, Visualization, and Integrated Discovery (DAVID version 2021, https://david.ncifcrf.gov/home.jsp) (Han et al., 2022). The 22 DEGs distracted from all 3 datasets were uploaded to DAVID, and *p* < 0.05 was identified as the critical threshold for significant enrichment. The GO term included the following three criteria: molecular function (MF), cell composition (CC), and biological process (BP).

### 2.4 PLP1 methylation analysis

The CpG islands around the PLP1 gene promoter were profiled by the UCSC Genome online tool (https://genome.ucsc.edu/). The DNA methylation data of the PLP1 gene was retrieved from the DiseaseMeth version 2.0 database (http://bio-bigdata.hrbmu.edu.cn/diseasemeth/) (Song et al., 2022). The RNA modification type of PLP1 was identified by m6A-atlas (Tang et al., 2021) (http://rnamd.org/m6a) and m5C-atlas (Ma et al., 2022) (http://rnamd.org/m5c-atlas/index.html). The possible m6a regulator of PLP1 was analyzed by the online tools m6a target (http://m6a2target.canceromics.org/).

### 2.5 Clinical data

A total of 48 patients of UFs were recruited for this study who underwent myomectomy or hysterectomy with a final histological diagnosis of uterine fibroids in Tongji Hospital from 2018–2020. 14 UFs-free individuals were considered a control group. The slices of normal myometrium tissue were difficult to obtain, especially for UFs patients who underwent myomectomy. We included the patients with single uterine prolapse (8/14) who underwent hysterectomy, or patients with cesarean section scar diverticulum (CDS) who were treated by hysteroscopy (6/14) as a comparable control. The normal myometrium tissue from the slices of the CDS patients was identified by pathologists and only the section of myometrium tissue was included for further IHC analysis. Exclusion criteria for all participants consisted of fibroid degeneration, leiomyosarcoma, adenomyosis, and other gynecologic or pelvic malignant disorders. Any women with complicated diseases, for example, metabolic disorders, hypertension, autoimmune diseases, and treated with hormones before surgery were excluded. The information of all patients was collected from electronic medical records in Tongji Hospital which contains age, myoma location (FIGO), the maximum diameter of fibroids, and previous history of pregnancies and surgery. All procedures of this study were approved by the Ethics Committee of Tongji Medical College, Huazhong University of Science and Technology (2022S068).

### 2.6 RT-qPCR

Total RNA was extracted from leiomyoma and normal myometrium tissue using RNA-easy Isolation Reagent (Vazyme, R701). Synthesis of cDNA was performed using the PrimeScript^™^ RT Master Mix (Takara, RR036A). Then, real-time PCR analyses (Vazyme, Q712-02) were carried out in triplicate for each sample. All gene expression was normalized to GAPDH. The expression levels were calculated using the 2-∆∆ Cq method (Livak and Schmittgen, 2001). The PCR primers were listed at supplemental Tabel1.

### 2.7 Western blot

All leiomyoma and normal myometrium tissue was lysed by RIPA buffer contended with 1% PMSG. Standard western blotting procedures were used (Liu C. et al., 2021). The primary antibody used PLP1 (Abcam, ab254363, 1:2000). Equal loading was confirmed using the glyceraldehyde-3-phosphate dehydrogenase (GAPDH) antibody (CST, 5174S, 1:2000). The appropriate anti-Rabbit HRP-linked secondary antibody (CST, 7074, 1:3000) was used.

### 2.8 Immunohistochemistry and hematoxylin and eosin stain

The section (4-μm thick) of paraffin-embedded leiomyoma and normal myometrium were deparaffinized and rehydrated using a series of graded xylene and alcohol. All slices used EDTA for antigen retrieval. After 1 h cooled, 10% H_2_O_2_ was used to quench endogenous peroxidase activity. Blocking was performed using goat serum for 30 min, RT. The primary antibody used PLP1 (Abcam, ab254363, 1:2000). The HRP labeled anti-Rabbit secondary antibody was used the following day. Finally, the slices were mounted with the coverslips using Permount TM Mounting Medium. And all adjacent slices were stained with hematoxylin and eosin (H&E) based on the basic protocol. The percentage of positive stained was conculcated as follows (Karpathiou et al., 2021): 0 = 0%, 1 = 0–25%, 2 = 26–50%, 3 = 51–75%, 4 = 76–100%. The intensity scoring was conducted as follows: 0 = no staining, 1 = weak, 2 = moderate, 3 = strong. The final scores of all sections were based on multiplying the percentage by intensity. [0] = negative expression; [1–3] = low expression; [4–12] = high expression.

### 2.9 Statistical analysis

All data were presented as the mean ± SD, and data generated *in vitro* were compared using Student’s t-tests. We performed χ2 test to explore the relationship between UFs and normal myometrium for categorical data. Receiver operating characteristic (ROC) analysis based on the IHC score of all cases was performed to evaluate the diagnostic value of PLP1. The optimal cutoff value in the ROC curve was set to the value that maximizes the Youden index. Youden’s index was defined as sensitivity + specificity—1. The statistical significance threshold was set at a *p*-value of <0.05. SPSS v21.0 (IBM, United States) and GraphPad Prism 8.0 (GraphPad, United States) were used for statistical analysis and figures preparation.

## 3 Results

### 3.1 Identification of differentially expressed genes between uterine fibroids and normal myometrium

**FIGURE 1:**
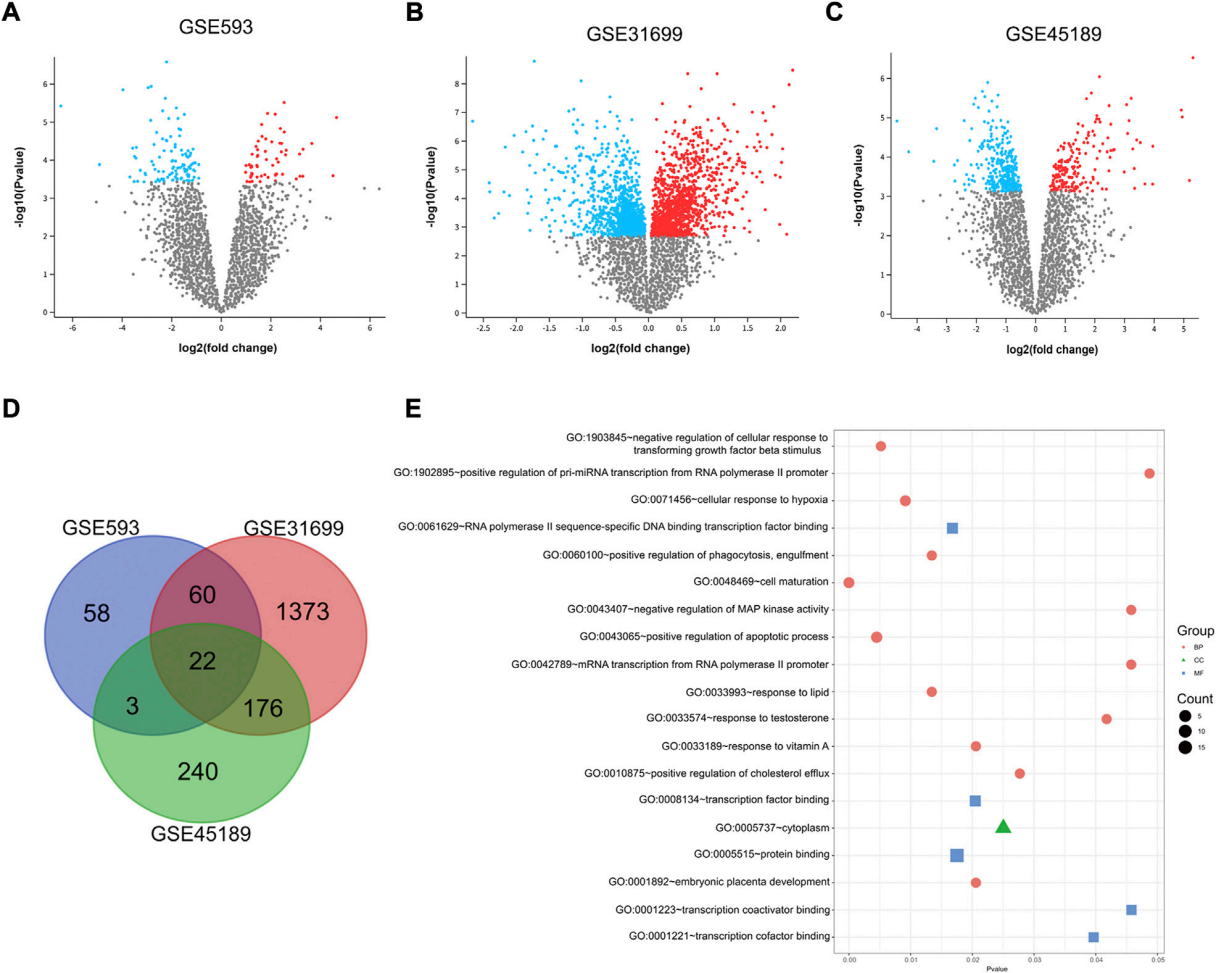
DEGs identified by GEO2R. Volcano plot of differentially expressed genes in GSE593 **(A)**, GSE31699 **(B)**, and GSE45189 **(C)** datasets. Up-regulation and down-regulation genes are marked with red and blue respectively. The criteria for a DEG are |log2FC|>1 and adjusted *p*-value < 0.05. **(D)** Venn diagram based on the DEGs from all 3 datasets, 22 genes were extracted. **(E)** Gene Ontology Enrichment analysis of 22 DEGs. *P* < 0.05 was identified as the critical threshold for significant enrichment. MF, molecular function, CC, cell composition, BP, and biological process.

A total of 163 DEGs were identified between UFs and normal myometrium in GSE593 data set by GEO2R analysis, including 58 upregulating genes and 105 downregulating genes. DEGs were analyzed from the gene expression profiles in GSE45189 and GSE31699 datasets respectively. Thereinto, 189 upregulated and 309 downregulated genes were identified by GEO2R analysis in GSE45189 dataset. As for GSE31699 dataset, 2060 DEGs were identified, which included 1129 upregulating genes and 931 downregulated genes. The DEGs of UFs and normal myometrium for each dataset was visualized in the corresponding volcano plots (Figures 1A–C). The DEGs from all datasets were identified by the Venn diagram (Figure 1 D), 22 DEGs were shown in Table 2.

**TABLE 2:** Gene symbol of 22 differentially expressed genes.

Gene symbol	Gene full name	FDR
KIF5C	kinesin family member 5C	4.652
PLP1	proteolipid protein 1	3.158
RAD51B	RAD51 paralog B	2.189
ZMAT3	zinc finger matrin-type 3	1.867
TYMS	thymidylate synthetase	1.795
NAV2	neuron navigator 2	1.403
CFLAR	CASP8 and FADD like apoptosis regulator	-0.91
PRKCH	protein kinase C eta	-1.07
ITGB4	integrin subunit beta 4	-1.351
MPP5	membrane palmitoylated protein 5	-1.379
GPC4	glypican 4	-1.408
TNFSF10	tumor necrosis factor superfamily member 10	-1.505
ADIRF	adipogenesis regulatory factor	-1.608
EPAS1	endothelial PAS domain protein 1	-1.634
GATA2	GATA binding protein 2	-1.709
CALCRL	calcitonin receptor like receptor	-1.947
ABLIM1	actin binding LIM protein 1	-2.04
ABCA8	ATP binding cassette subfamily A member 8	-2.644
PPARG	peroxisome proliferator activated receptor gamma	-2.74
SPTBN1	spectrin beta, non-erythrocytic 1	-3.376
TM4SF1	transmembrane 4 L six family member 1	-3.498
DUSP1	dual specificity phosphatase 1	-4.915

### 3.2 Analysis of Gene Ontology Enrichment

The GO enriched terms were analyzed by DAVID database. The results showed that DEGs between UFs and normal myometrium of all 3 datasets were mainly enriched in cell maturation, regulation of the apoptotic process, cellular response to hypoxia, and response to testosterone in the biological process. Protein binding was the most enrichment term in the molecular function criterion. Considering the cell composition criterion, the results showed that DEGs mainly concentrated on the cytoplasm. All terms of GO analysis are presented in Figure 1E.

### 3.3 Identify the hub gene

**FIGURE 2:**
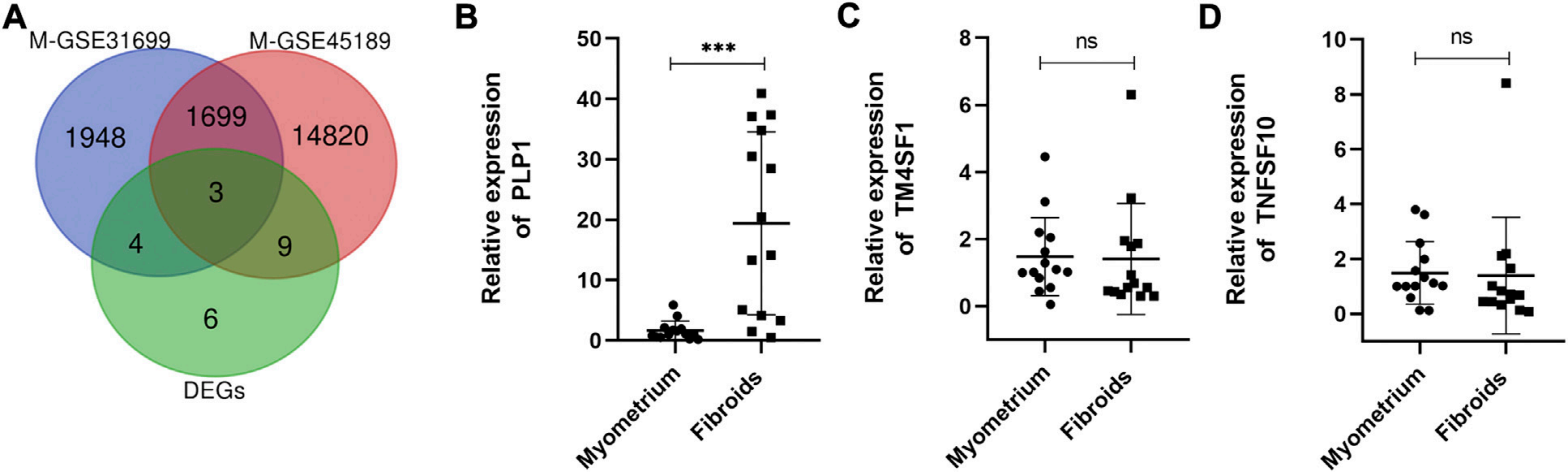
Identification of the hub gene. **(A)** The co-expressive differentially expressed genes in differently mRNA expression profiles (from GSE593, GSE31699, and GSE45189 datasets) and genome-wide DNA methylation genes (from GSE31699, GSE45189 datasets) by Venn diagram. M-GSE31699, M-GSE45189 indicated the differential DNA methylation genes. **(B–D)** The relative mRNA expression of TM4SF1, TNFSF10, and PLP1 by RT-qPCR. *n* = 14/groups. ^***^ *p* < 0.001. ns no statistical significance.

Epigenomic aberrations, especially DNA methylation have been identified as one of the main mechanisms for UFs pathogenesis (Mlodawska et al., 2022). Over the years, literature has reported that aberrant DNA methylation occurs throughout the genome in UFs (Islam et al., 2013; Sato et al., 2014; Sato et al., 2016; Sato et al., 2019; Maekawa et al., 2022), accompanied by mRNA expression discrepancy, demonstrating that aberrant gene expression caused by aberrant DNA methylation plays a key role in the pathogenesis of UFs. The datasets included in this study, GSE31699 and GSE45189, provided data about genome-wide DNA methylation of UFs (Navarro et al., 2012; Maekawa et al., 2013). The differential DNA methylation genes were analyzed by GEO2R. The volcano plots of the hypermethylation/hypomethylation genes were presented in the supplemental figure. To investigate the accurate genes with aberrantly DNA promoter methylation, we drew the Venn diagram as followed (Figure 2A). The differential DNA methylation genes in both GSE31699 and GSE45189 datasets and the 22 DEGs extracted from all 3 datasets were analyzed, and three hub genes were found (TM4SF1, TNFSF10, PLP1). The RT-qPCR was implemented to verify the gene expression. Among them, PLP1 was overexpressed in UFs tissue (Figures 2B–D).

### 3.4 The expression of PLP1 might be regulated by both DNA methylation and RNA modification

Multiple CpG islands were present in Supplemental Figure S2. Then, we found that the PLP1 transcripts were methylated to varying degrees in uterine carcinosarcoma and uterine corpus endometrial carcinoma (Supplemental Figure S2). Interestingly, we discovered that PLP1 was linked to N6-methyladenosine (m6A) modification. Metagene analysis of m6A indicating modification of PLP1 in 3′UTR gene region in the human embryonic stem cells (ESC). The RNA binding protein and binding region were present in Supplemental Table S2. We curated and analyzed a set of 5 acknowledged m6A regulators of PLP1 (4 readers and 1 writer). The detailed descriptions of m6A regulators were present in Supplemental Table S3.

### 3.5 Baseline characteristics of the patients

To verify the expression of PLP1 in UFs tissue, we collected tissue samples from UFs patients. A total of 48 UFs patients from Tongji Hospital who underwent abdominal surgery caused by uterine fibroids (UFs) and 14 UFs-free individuals were included in this study. The mean age of all participants enrolled in this study is approximately 45, of which the age of UFs patients is 42 and UFs-free participants is 44. There was no statistical difference in age, number of pregnancies, and whether previous abdominal surgery was performed between these two groups. The basic characteristics of all participants were shown in Table 3. The additional fibroid characteristics of UFs patients were summarized in Table 4. The mean maximum diameter of leiomyoma in this study is 6.4 cm. The mean size of UFs in this study was relatively large because all those fibroids were detected for surgical reasons. Myomectomy was operated on in 79.2% (38/48) of patients while the rest of the patients (20.8%, 10/48) underwent a hysterectomy. Notably, 12.5% (6/48) of patients had a previous myomectomy.

**TABLE 3:** Baseline characteristics of all participants.

Characteristic		UFs (n = 48)	UFs-free (n = 14)	*p*-value
Age (mean ± SD; range)		42.10 ± 7.23; 27–53	44.07 ± 11.22; 29–61	0.435
No. of pregnancies (mean ± SD; range)		2.17 ± 1.49; 0–5	2.85 ± 1.28; 0–5	0.127
Previous abdominal surgery				
	yes	23	9	0.281
	no	25	5	

Note: SD, standard deviation; UFs, uterine fibroids.

**TABLE 4:** Fibroid characteristics in participants with fibroids (n = 48).

Parameters		No. cases	%
Location			
	Anterior	16	33.3
	Posterior	12	25.0
	Lateral	6	12.5
	Fundal	10	20.8
	others (broad ligament, cervix *etc.*)	4	8.3
Maximum diameter			
	mean		
	<5	6	12.5
	5–8	37	77.1
	>8	5	10.4
Surgery type			
	myomectomy	38	79.2
	hysterectomy	10	20.8
Previous myomectomy			
	Yes	6	12.5
	No	42	87.5

### 3.6 Elevated PLP1 expression in UFs tissue

**FIGURE 3:**
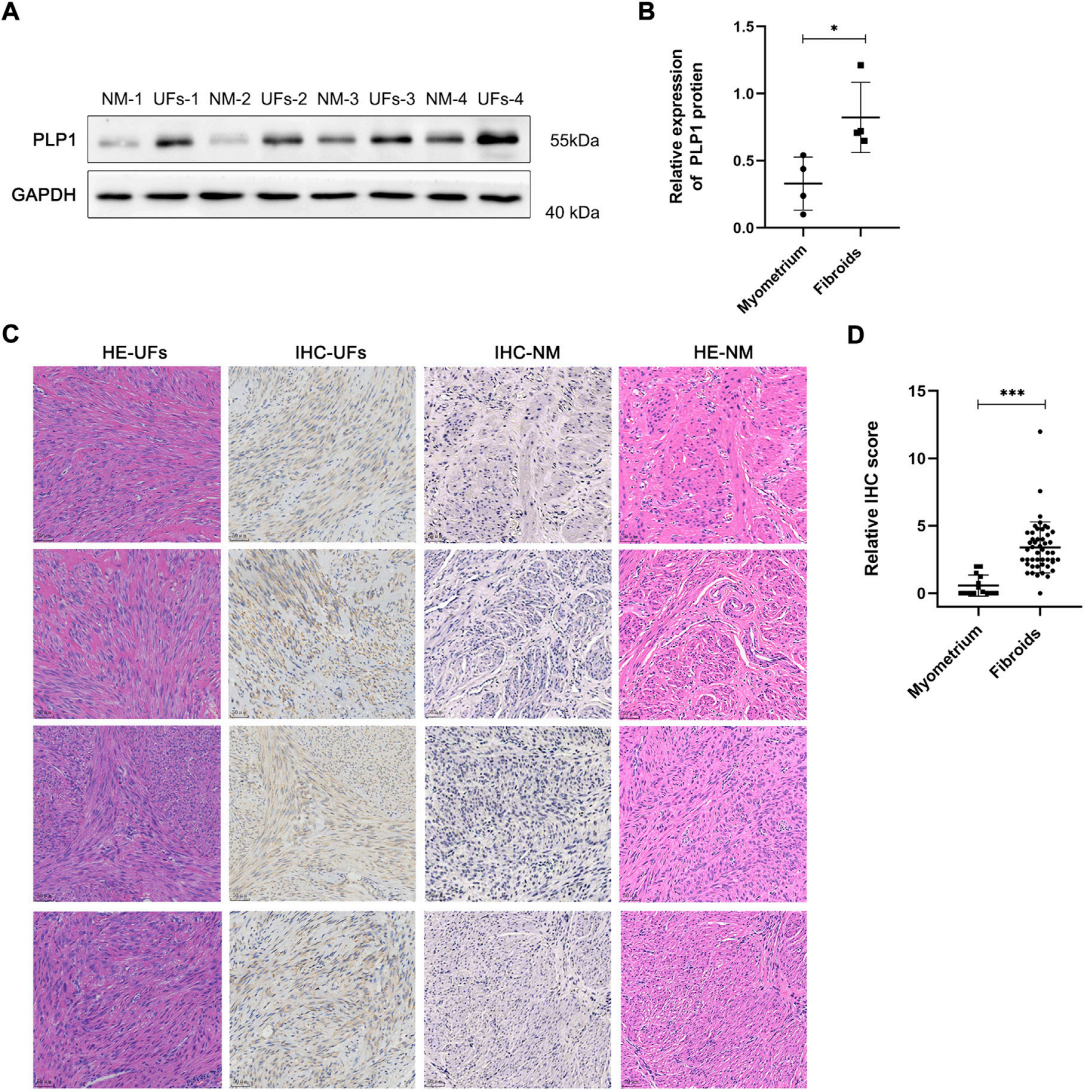
PLP1 expression in UFs tissue **(A)** The PLP1 protein expression of tissue from fibroids and normal myometrium was assessed by Western blot. **(B)** The relative expression of PLP1 is based on the gray value of Western blot. N = 4/groups. ^*^ *p* < 0.05. **(C)** Immunohistochemical and corresponding hematoxylin and eosin stains results of fibroids and normal myometrium tissue (magnification 200). IHC, immunohistochemical, HE, hematoxylin and eosin stains, UFs, Uterine Fibroids, NM, Normal Myometrium. **(D)** Relative IHC score. UFs, n = 48. NM, n = 14. ^***^ *p* < 0.001.

The qRT-PCR and Western blot results showed PLP1 was overexpressed in UFs compared with that in normal myometrium (Figure 2B, Figures 3A,B). In addition, IHC staining of PLP1 was performed in UFs and paired normal myometrium (Figure 3C). The corresponding H&E-stained sections were also shown in Figure 3C to illustrate the histological characteristics of UFs. PLP1 was upregulated in UFs compared with that in normal myometrium tissue. The relative IHC score based on all cases demonstrated the expression of PLP1 significantly increased in UFs (Figure 3D).

### 3.7 ROC curve analysis

**FIGURE 4:**
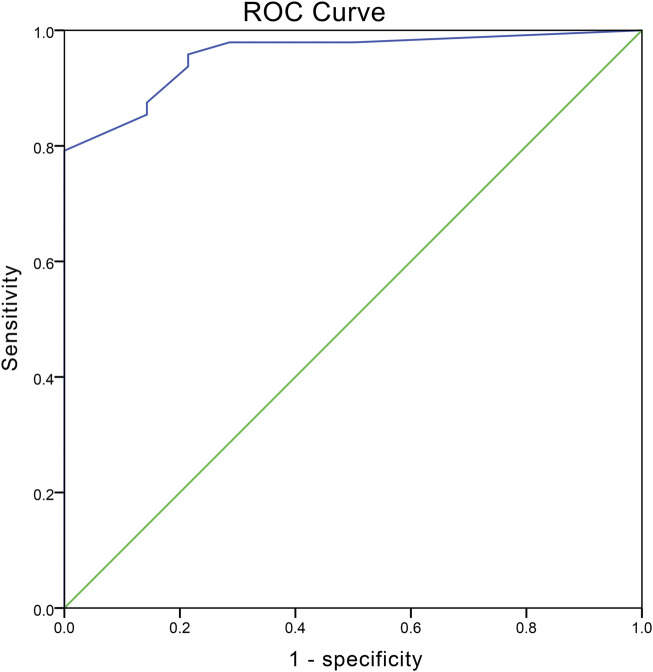
ROC curve depicting the diagnostic value of PLP1. The values of AUC, optimum cutoff, sensitivity, and specificity are 0.956 (*p* < 0.001), 2.069, 79.2% and 100%, respectively.

The diagnostic value of PLP1 of uterine fibroids was determined by the ROC curve which was constructed by the IHC score (Figure 4). The area under the ROC curve (AUC) was 0.956 with *p* < 0.005. The results showed the cutoff value was 2.069 with a sensitivity of 79.2% and a specificity of 100%, suggesting PLP1 presented high diagnostic accuracy of UFs.

## 4 Discussion

Uterine fibroids, formed by the proliferation of smooth muscle cells, are one of the most common benign tumors in women of reproductive age (Styer and Rueda, 2016). Numerous studies have demonstrated the potential biomarker for the diagnosis and surveillance of UFs, but the efficacies were still unclear (Levy et al., 2013). In this study, comprehensive bioinformatics methods were used to verify the biomarker as well as to investigate the possible molecular mechanism underlying the development of UFs.

We analyzed the DEGs of 3 datasets, including GSE593, GSE45189, and GSE31699 datasets, under the same criteria using GEO2R. 22 DEGs were found between UFs and normal myometrium tissue samples. The DEGs were mainly enriched in cell maturation and regulator of apoptotic. It is well known that apoptosis is one of the key regulators of fibroid growth, and the dysregulation of apoptotic pathways may contribute to the development of UFs (Okolo, 2008). Another main enrichment in the biological process was the cellular response to hypoxia. Leiomyoma grows in the hypoxia microenvironment, and such environment may lead to the formation of UFs (Zhou et al., 2011; Tal and Segars, 2014). The hypoxia-inducible factor-1protein was also overexpressed in UFs tissue compared with myometrium (Miyashita-Ishiwata et al., 2022a; b). Response to testosterone was also enriched in our analysis. Fujimoto *et al.* reported that testosterone increased after treatment with estradiol dipropionate in leiomyoma, while it not occurred in the myometrium, which indicated that testosterone might participate in the biological process of UFs (Fujimoto et al., 1994). Nonetheless, Ke LQ *et al.* failed to find reliable evidence to prove the effectiveness of danazol, a synthetic isoxazole derivative chemically related to 17-ethinyl testosterone, in UFs in clinical trials (Ke et al., 2009). Although there is still an uncertain conclusion on the response of testosterone in UFs, the aberrant activities of this process might impact the development of UFs, which is consistent with our enrichment analysis. Most of the DEGs were enriched in protein binding for molecular function (19/22) based on our results.

As one of the well-studied epigenomic processes in mammals (Bestor, 2000), DNA methylation was considered as a potential mechanism in the pathology of UFs. Many aberrantly hypermethylation/hypomethylation genes were detected in by genome-wide DNA methylation assays (Li et al., 2003; Yamagata et al., 2009; Navarro et al., 2012; Islam et al., 2013; Maekawa et al., 2013; Carbajo-García et al., 2022), and numerous genes were validated to participate in the developmental progress of the UFs *in vitro* experiments. SATB homeobox 2 and neuregulin 1 were proved to be the upregulated hypermethylated genes involved in the pathogenesis of uterine leiomyoma by activating the WNT/β-catenin and TGF-β pathways (Sato et al., 2019). Shimeng Liu *et al.* sorted cells from UFs tissue into stem cell-like cells and revealed that most of the stem cells in UFs were hypermethylated. Meanwhile, tumor growth was suppressed when administered the hypomethylating drug, 5′-Aza (Liu et al., 2020; Liu S. et al., 2021). The methylation condition of mediator complex subunit 12 (MED12), one of the most widely reported somatic mutation genes in UFs (Mäkinen et al., 2011), also could separate the UFs from myometrium on account of the aberrantly clustering molecular pathways based on the MED12 methylation-induced DEGs (Maekawa et al., 2022). All these facts combined with the genome-wide DNA methylation profile of UFs suggested that methylation was the vital epigenetic mechanism in UFs and the significant DEGs between UFs and myometrium might be induced by the local changes of DNA methylation at genome loci. To identify the key genes in this progress, we next combined the DEGs extracted above with the different DNA methylation conditions genes in both GSE45189 and GSE31699 datasets. Consequently, TM4SF1, TNFSF10, and PLP1 were identified, in which only PLP1 was significantly upregulated as verified by RT-qPCR.

PLP1 is the most abundant protein of myelination (Eng et al., 1968; Norton and Poduslo, 1973; Wight, 2017), and the mutation of PLP1 can lead to the X-chromosome-linked leukodystrophy Pelizaeus–Merzbacher disease (Elitt et al., 2020). PLP1 has been widely reported in the formation of the central nervous system while the aberrant expression of PLP1 in various malignant tumors was identified by the bioinformatic analysis (Li et al., 2017). Remarkably, the high level of PLP1 in primary colorectal cancer patients presents poorer overall survival times than those with low expression levels (Han et al., 2020). Although the underlying mechanism is unclear, PLP1 was still considered a potential biomarker in other diseases other than only in nervous system lesions (Khalaf et al., 2022). PLP1 was considered as the hypomethylation and transcriptionally upregulated genes in leiomyoma based on the genome-wide DNA methylation and mRNA expression analysis (Navarro et al., 2012). The results of this present study showed that the PLP1 was over-expressed in UFs tissue based on the comprehensive analysis of irregular methylation genes and DEGs in fibroids. Then the validation was conducted at both mRNA and protein levels based on the tissue samples from leiomyoma patients. To the best of our knowledge, it is the first time to validate the dysregulation of PLP1 in benign tumors. Our result suggested that the hypomethylation of PLP1 might be involved in the pathophysiology of UFs, but further experiments still need to implement. Moreover, overexpressed PLP1 exhibited major oxidative phosphorylation deficits (Wight, 2017) and the down-regulation of oxidative phosphorylation aggravates therapeutically adverse tumor hypoxia (Ashton et al., 2018). According to our result that the 22 DEGs including PLP1 were enriched in hypoxia, we constructed the posits that PLP1 might participate in the hypoxia program to onset the fibroids. However, the underlying mechanisms were speculated through different tissue types, which should be further investigated in UFs.

To evaluate the diagnostic meaning of PLP1 expression in UFs, we drew the ROC curve based on the calculation of the IHC score, indicating that PLP1 expression can be a convincing biomarker for UFs with the AUC, sensitivity, and specificity of 0.956, 79.2%, and 100%, respectively. However, the limitation of this ROC analysis is based on IHC score only, lacking clinical utility. The specificity analysis was limited by the fact that PLP1 expression tissue samples were derived only from UFs and normal myometrium, although it was credible.

## 5 Conclusion

In summary, we obtained 22 DEGs in UFs *via* bioinformatical analysis and identified PLP1 as the core gene by the combined analysis of genome-wide DNA methylation profiles and 22 DEGs. The over-expression of PLP1 in UFs tissue was validated in both mRNA and protein levels for the first time. Our findings indicated that PLP1 is a potential diagnostic biomarker of the UFs.

## Data Availability

The original contributions presented in the study are included in the article/Supplementary Material, further inquiries can be directed to the corresponding authors.
