# Supplementary material for: PLP1 may serve as a potential diagnostic biomarker of uterine fibroids
Source: Front Genet. 2022 Oct 31;13:1045395. doi: 10.3389/fgene.2022.1045395 (PMC9662689; doi:10.3389/fgene.2022.1045395)
Supplement: Supplementary file 1 [file Table1.DOCX]

| **SUPPLEMENTAL TABLE 1** | |
| --- | --- |
| **Genes** | **Primers and probes (5’ to 3’)** |
| PLP1 | F: GCTAGGACATCCCGACAAGT |
|  | R: GGTGGTCCAGGTGTTGAAGTA |
| TM4SF1 | F: CCAATGGGGAAACAAAGT |
|  | R: CCAATGAAGACAAATGCTG |
| TNFSF10 | F: AGTGGCATTGCTTGTTTC |
|  | R: AGCCTTTTCATTCTTGGA |
| GAPDH | F: AGAAGGCTGGGGCTCATTTG |
|  | R: AGGGGCCATCCACAGTCTTC |
