# Supplementary figures and images for: PLP1 may serve as a potential diagnostic biomarker of uterine fibroids
Source: Front Genet. 2022 Oct 31;13:1045395. doi: 10.3389/fgene.2022.1045395 (PMC9662689; doi:10.3389/fgene.2022.1045395)

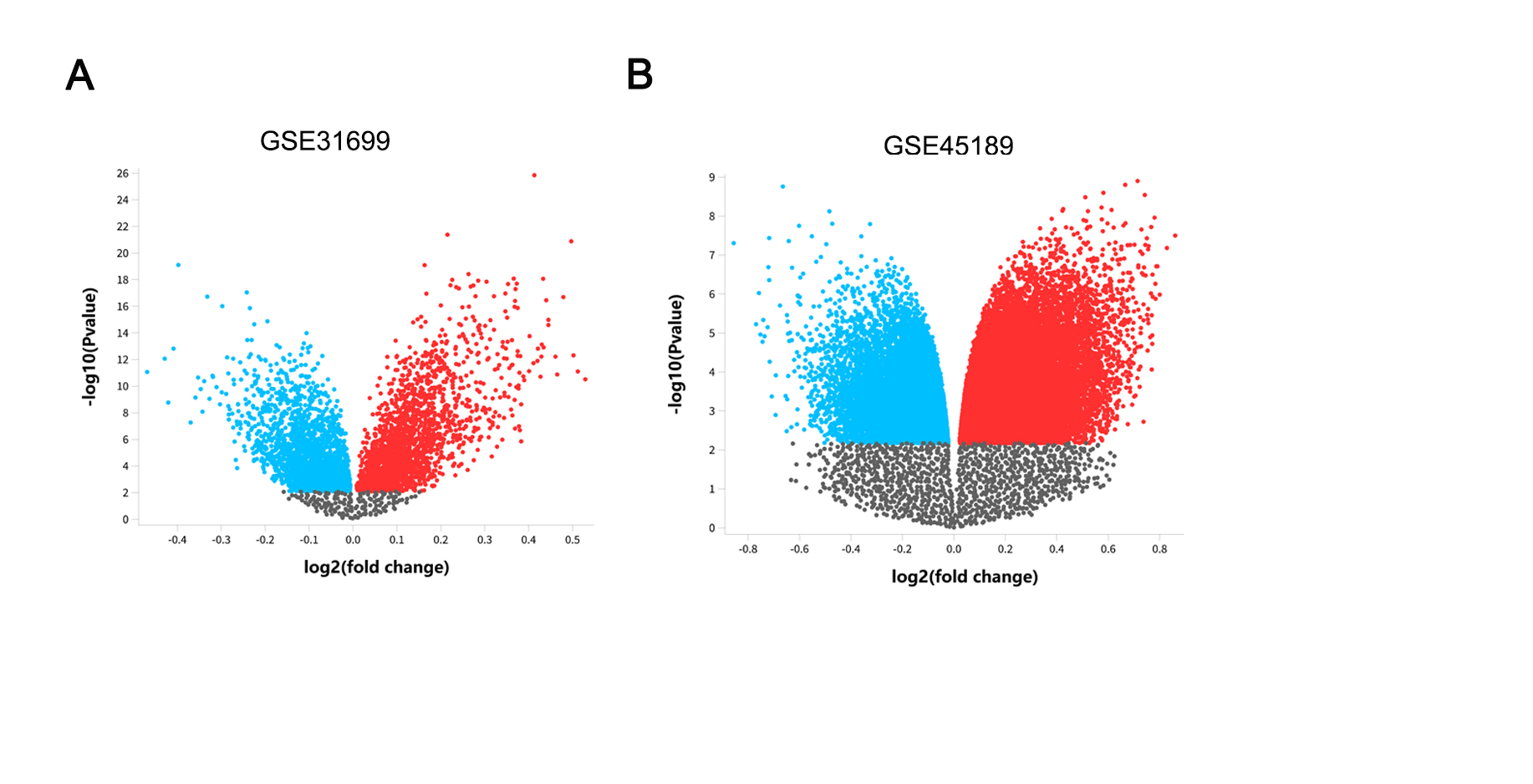

Supplement: Supplementary file 2 [file Image1.JPEG]

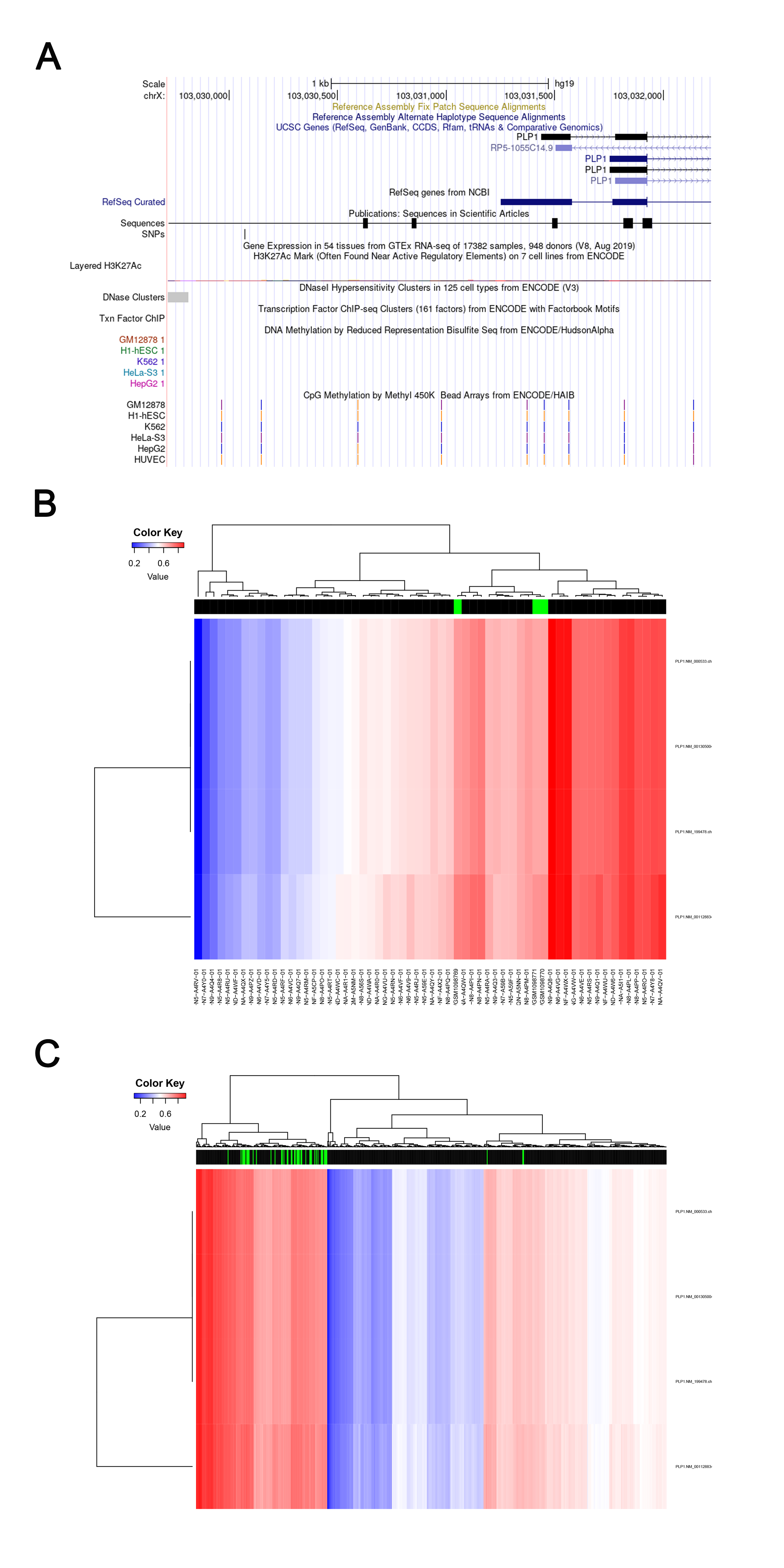

Supplement: Supplementary file 3 [file Image2.JPEG]
