# Supplementary material for: PLP1 may serve as a potential diagnostic biomarker of uterine fibroids
Source: Front Genet. 2022 Oct 31;13:1045395. doi: 10.3389/fgene.2022.1045395 (PMC9662689; doi:10.3389/fgene.2022.1045395)
Supplement: Supplementary file 4 [file Table2.DOCX]

| **SUPPLEMENTAL TABLE 2** The details of RNA banding protein of PLP1 | | | | | |
| --- | --- | --- | --- | --- | --- |
| **RBP** | **Database** | **Study** | **Site** | **Binding Region** | **Technique** |
| FUS | POSTAR2 | GSE43308 | 3' UTR | chrX:103792391-103792411 | MAZTER-seq |
|  |  | GSM1060382 |  |  |  |
|  |  |  |  |  |  |
| MBNL2 | POSTAR2 | GSE68890 | 3' UTR | chrX:103791751-103791791 | MAZTER-seq |
|  |  | GSM1685415 |  |  |  |
|  |  |  |  |  |  |
| MBNL2 | POSTAR2 | GSE68890 | 3' UTR | chrX:103791771-103791791 |  |
|  |  | GSM1685414 |  |  |  |
|  |  |  |  |  |  |
| MBNL2 | POSTAR2 | GSE68890 | 3' UTR | chrX:103791771-103791791 |  |
|  |  |  |  |  |  |
| FUS | POSTAR2 | GSE40653 | 3' UTR | chrX:103791611-103791771 | MAZTER-seq |
|  |  | GSM998875 |  |  |  |
|  |  |  |  |  |  |
| MBNL2 | POSTAR2 | GSE68890 | 3' UTR | chrX:103791751-103791791 |  |
|  |  | GSM1685415 |  |  |  |
|  |  |  |  |  |  |
| FUS | POSTAR2 | GSE40653 | 3' UTR | chrX:103791611-103791771 | m6A-REF-seq |
|  |  | GSM998875 |  |  |  |
|  |  |  |  |  |  |
| TARDBP | POSTAR2 | E-MTAB-530 | 3' UTR | chrX:103791644-103791665 |  |
|  |  | ERR039849 |  |  |  |
|  |  |  |  |  |  |
| FUS | POSTAR2 | GSE40653 | 3' UTR | chrX:103790792-103790912 | MAZTER-seq |
|  |  | GSM998875 |  |  |  |
|  |  |  |  |  |  |
| FUS | POSTAR2 | GSE40653 | 3' UTR | chrX:103790532-103790752 | MAZTER-seq |
|  |  | GSM998875 |  |  |  |
