# Supplementary material for: PLP1 may serve as a potential diagnostic biomarker of uterine fibroids
Source: Front Genet. 2022 Oct 31;13:1045395. doi: 10.3389/fgene.2022.1045395 (PMC9662689; doi:10.3389/fgene.2022.1045395)
Supplement: Supplementary file 5 [file Table3.DOCX]

| **SUPPLEMENTAL TABLE 3** The detailed descriptions of m6A regulators | | | | | | | | | |
| --- | --- | --- | --- | --- | --- | --- | --- | --- | --- |
| **Species** | **Cell lines** | **Class** | **WER name** | **WER detail** | **Target gene** | **Target site** | **Interaction** | **Method** | **Down-stream effects** |
| Human | HEK293T | Reader | IGF2BP1 | insulin like growth factor 2 mRNA binding protein 1 | PLP1 | intron (NM_001305004, intron 1 of 6)/intron (NM_001305004, intron 1 of 6)/3' UTR (NM_199478, exon 7 of 7) | Protein-RNA | CLIP-seq | No evidence |
| Human | HEK293T | Reader | IGF2BP2 | insulin like growth factor 2 mRNA binding protein 2 | PLP1 | 3' UTR (NM_199478, exon 7 of 7)/exon (NM_199478, exon 6 of 7) | Protein-RNA | CLIP-seq | No evidence |
| Human | HEK293T | Reader | YTHDC1 | YTH domain containing 1 | PLP1 | Intergenic | Protein-RNA | CLIP-seq | No evidence |
| Human | HEK293T | Reader | YTHDF1 | YTH N6-methyladenosine RNA binding protein 1 | PLP1 | Intergenic | Protein-RNA | CLIP-seq | No evidence |
| Human | MDA-MB-231 | Writer | METTL3 | methyltransferase like 3 | PLP1 | / | Protein-RNA | RIP-seq | No evidence |
